# Autosomal dominant polycystic kidney disease: recent advances in clinical management

**DOI:** 10.12688/f1000research.9045.1

**Published:** 2016-08-18

**Authors:** Zhiguo Mao, Jiehan Chong, Albert C. M. Ong

**Affiliations:** 1 Kidney Institute of CPLA, Division of Nephrology, Changzheng Hospital, Second Military Medical University, Shanghai, China; 2 Kidney Genetics Group, Academic Nephrology Unit, University of Sheffield Medical School, Sheffield, UK; 3 Sheffield Kidney Institute, Sheffield Teaching Hospitals NHS Foundation Trust, Sheffield, UK

**Keywords:** autosomal dominant polycystic kidney disease, ADPKD, PDK1, PDK2, clinical management

## Abstract

The first clinical descriptions of autosomal dominant polycystic kidney disease (ADPKD) go back at least 500 years to the late 16 ^th^ century. Advances in understanding disease presentation and pathophysiology have mirrored the progress of clinical medicine in anatomy, pathology, physiology, cell biology, and genetics. The identification of *PKD1 *and *PKD*2, the major genes mutated in ADPKD, has stimulated major advances, which in turn have led to the first approved drug for this disorder and a fresh reassessment of patient management in the 21 ^st^ century. In this commentary, we consider how clinical management is likely to change in the coming decade.

## Introduction

Autosomal dominant polycystic kidney disease (ADPKD) is the most common inherited renal disease worldwide and the fourth most common cause of end-stage renal disease (ESRD) ^1^. It is a significant economic health burden to societies, and annual costs of providing renal replacement therapy (RRT) within the European Union (EU) are estimated at 1.2 billion euros. The median age of ESRD for patients with ADPKD within the EU is 58 years and has shifted only slightly over the past two decades (1991–2010) with the acceptance of older patients onto RRT programmes ^2^. The management of ADPKD, long considered an untreatable disease, is undergoing a major paradigm shift with regulatory approval of the first effective drug for delaying disease progression. In this review, we consider some of the major recent advances that have led to this shift in clinical management.

## Prevalence, natural history, and clinical presentation

Estimates of the prevalence of ADPKD vary widely depending on the population studied, methodology used, and local screening policy ^1^. Within the EU, ADPKD is now considered to fulfil the definition of a rare disease (less than 1 in 2,000 affected) and has implications for health policy and funding ^3^. This issue is still debated (asymptomatic cases may be undiagnosed without comprehensive screening of at-risk family members), but the definition probably applies to symptomatic cases.

**Figure 1.**
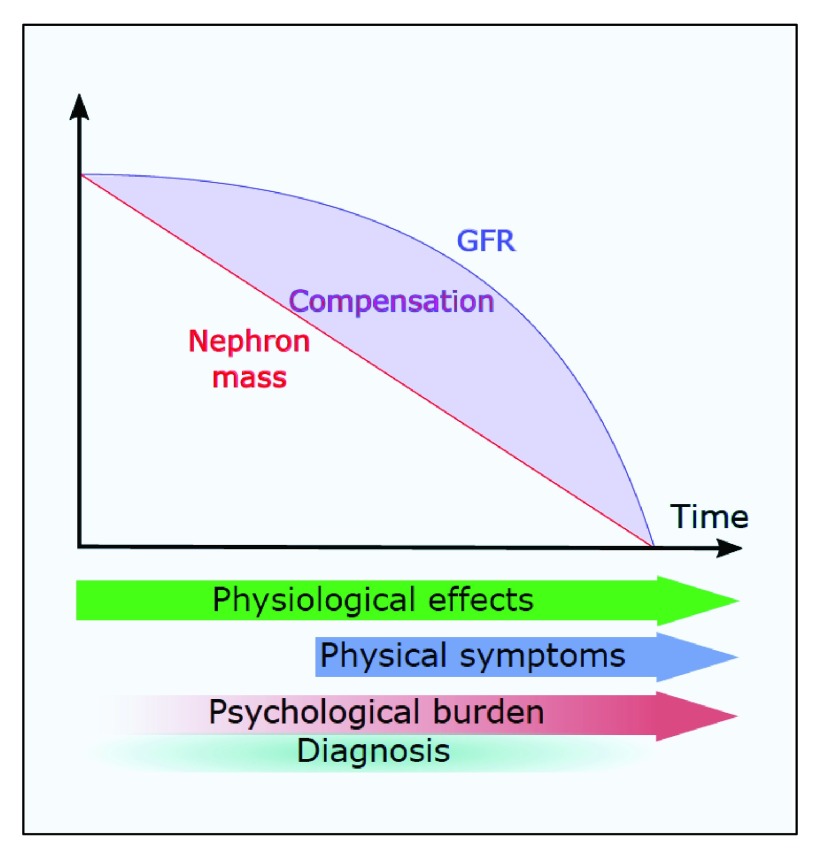
The natural history of autosomal dominant polycystic kidney disease as depicted by renal function decline as well as the onset of physical and psychological symptoms. Reductions in total nephron mass are masked by compensatory changes in glomerular filtration rate (GFR) such that total GFR remains apparently normal for many years until compensation fails. Subclinical physiological changes are detectable from the earliest stages of disease (for example, in children), whereas clinical symptoms usually occur later. The psychological burden of having the diagnosis made is generally underestimated.

The diagnostic uncertainty, especially early in disease, reflects the natural history of the condition with a long phase of stable renal function (glomerular filtration rate, or GFR) followed by a steep linear decline late in the course of disease ( Figure 1). The latent phase of disease, especially in the early years, masks subtle changes in kidney physiology and the often silent but progressive expansion in kidney size. Studies in children with ADPKD reveal glomerular hyperfiltration, microalbuminuria, loss of urinary concentration, and loss of the normal diurnal blood pressure rhythm as early subclinical manifestations ^4– 7^.

Depending on the health system, patients may opt to undergo cascade screening or may present with typical symptoms (urinary tract infection, macroscopic haematuria, renal pain, or kidney stones) at different ages ^8^. A significant proportion of at-risk individuals remain asymptomatic, being diagnosed incidentally or through the detection of hypertension or reduced GFR. Often ignored are psychological symptoms reflective of concern and loss of hope about the future (risk of inheriting the disease, uncertainty about the timing, and options for delaying ESRD) as well as inter-personal issues (guilt about transmitting the condition to the next generation) which impact individual quality of life ^9^.

## Diagnostic modalities and genetic testing

Mutations in two genes, *PKD1* and *PKD2*, have been found in over 90% of patients with ADPKD, and no mutations have been detected in 8% to 10%. Evidence of a third gene, *GANAB*, was recently reported, although this accounts for only a small number of these *PKD1* and *PKD2* mutation-negative patients ^10^. In these patients, the renal phenotype was mild and the extent of liver disease was highly variable. It seems likely that incorrect ascertainment ^11^, mutations in promoter regions, or mutations in other unidentified genes could account for the rest.

**Figure 2.**
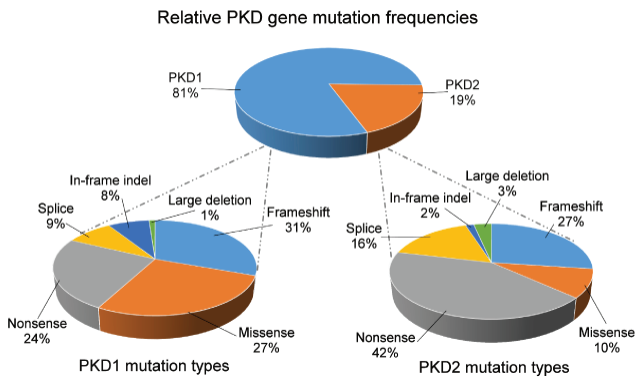
Frequency and type of *PKD1* and *PKD2* mutations from the PKD mutation database. All mutation types have been reported for both genes. The relative infrequency of missense mutations and in-frame insertions or deletions for *PKD2* could reflect the under-diagnosis of these patients present in the general population. The PKD mutation database is available at http://pkdb.mayo.edu/index.html (accessed 22 April 2016).

In large published series, the percentage of patients with *PKD2* mutations ranges from 10.5% to 22% ^12– 15^. This is very likely to reflect criteria for patient referral and selection, including their stage of kidney function. In recent data obtained from the PKD mutation database (accessed 22 April 2016), 81% and 19% of curated patients had *PKD1* and *PKD2* mutations, respectively ( Figure 2). Although all mutation types have been reported for both genes, it is striking that only 10% of *PKD2* mutations were missense (compared with 27% of *PKD1*). This could reflect the under-diagnosis of milder asymptomatic cases of PKD2 present in the general population.

Although the two ADPKD proteins have been shown to function as a heterodimeric complex, patients with PKD2 have milder disease and the median age of ESRD is between 20 and 25 years later than that for PKD1 for reasons that are still unclear ^16^. This has contributed to diagnostic uncertainty, especially when radiological methods were used in younger patients (under 40 years). Newer ultrasound diagnostic criteria have been published which extend the previous criteria (Ravine *et al*.) restricted to those at 50% risk of inheriting *PKD1* to those with *PKD2* or without a positive family history ^17^. In addition, magnetic resonance imaging criteria applying to those with a negative or uncertain family history have been published ^18^.

Where diagnostic uncertainty remains, mutation analysis for both genes is becoming routine despite the technical challenge of analysing *PKD1*. Within the National Health Service (UK), testing is currently restricted to several patient groups for whom diagnostic certainty is clinically helpful. These include the need to exclude risk in potential living related kidney donors ^19^, those with atypical disease or a negative family history, rare cases of very early onset disease (less than 1%) ^20^ in which the recurrence rate is high for subsequent pregnancies, and in pre-natal or pre-implantation diagnosis. Traditional Sanger sequencing is being rapidly replaced by newer approaches using next-generation sequencing (NGS) techniques. The first uses long-range polymerase chain reaction to selectively amplify the *PKD1* transcript (and not those arising from the six *PKD1* homologous genes, or HGs) ^21^. The second has used a bioinformatics approach to distinguish sequence reads from genomic DNA unique to *PKD1* from the HG loci ^22^. As the cost of testing falls, it is likely that these NGS methods will transform the use of testing for both diagnostic and prognostic (see below) purposes. A list of laboratories world-wide offering mutation analysis for *PKD1* and *PKD2* can be found on the GeneTests website ( https://www.genetests.org/disorders/?disid=78527&ps=chld and https://www.genetests.org/disorders/?disid=78530&ps=chld).

## Prognostic prediction and scores

The known individual phenotypic variability of disease and recent availability of a potential effective treatment have stimulated efforts to derive an accurate prognostic score that could be clinically useful. Two main prognostic models have been proposed. The Mayo Classification relies on age-banded height-adjusted total kidney volume (TKV) ^23^, whereas the PROPKD score ^24^ relies on genotype and the age of onset of clinical symptoms. Although the two scores have not been directly compared, they have their relative merits. The Mayo Classification distinguishes two classes of disease (1 typical and 2 atypical) on the basis of a retrospective analysis of a historical cohort of 590 patients seen at a single centre ^23^. Class 1 could be further subdivided into five subclasses (A to E) on the basis of the measured historical rate of kidney growth (on serial magnetic resonance or computed tomography), which in turn correlated with the rate of estimated GFR (eGFR) decline in the test population. This simple classification was then shown to have predictive value in both internal (n = 162) and external (n = 173, Consortium for Radiologic Imaging Study of PKD or CRISP) validation cohorts. Patients in class 1C–E were shown to have more rapidly progressive disease (>3 ml/min per year) than those with class 1A and B. The PROPKD score combined genotype, gender, and an age cut-off (< or >35 years) of onset of urinary symptoms or hypertension on the basis of a cross-sectional study of the Genkyst cohort, a population of 1,341 patients from Brittany ^24^. On the basis of these criteria, the authors propose three groups of patients with low, intermediate, or high risk for progression to ESRD.

The advantage of the Mayo Classification is the ability to predict prognosis on the basis of a single TKV measurement. The authors also propose that TKV can account for individual differences related to genotype as well as non-allelic factors. This study was based on a population with preserved initial eGFR (median 75 ml/min per 1.73 m ^2^) with a low incidence of ESRD (22%) and has the advantage of studying patients at earlier stages of disease ^23^. However, whereas the majority of patients (>85%) remained within the same subclass over time, a number of 1A–D patients (13.4% of 284) moved to the higher subclass over time whereas a similar number of 1B–E patients (13.1% of 282) moved to the lower subclass. The Genkyst cohort was enriched for patients who had reached ESRD (44.6% versus 22.3%) and was therefore older (54.7 versus 44 years of age) than the Mayo cohort ^24^. Clinical scoring relied on a cut-off of 35 years and therefore could not be applied consistently to younger patients without symptoms. It should be noted that although these scores can distinguish groups or classes within populations, there was significant variability within the same groups, limiting precision for predicting individual prognosis ^15, 23, 24^. Further refinement will be necessary to improve precision for the individual patient and probably will involve a combination of imaging, genetic, and clinical scoring ^1^.

For imaging, issues of access, speed, and accuracy of analysis ^25, 26^ as well as harmonisation of accepted standards at a national and international level will be needed. For genotype-based scoring, further refinement of the ‘strength’ of non-truncating mutations through bioinformatics ^15^, family studies ^14^, or experimental approaches ^27, 28^ could increase its predictive value. The added value of other novel biomarkers ^29– 31^ to improve the performance of both models will need to be formally tested.

## Treatment options

**Figure 3.**
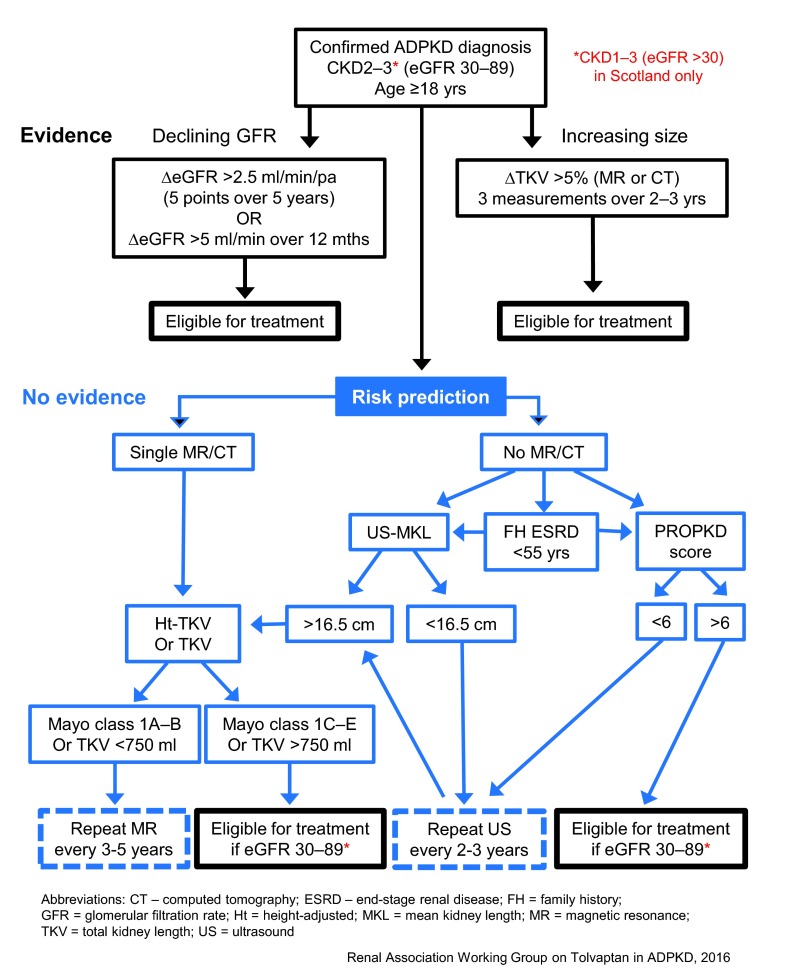
Recommended decision pathway for UK patients considered for treatment with tolvaptan according to Renal Association guidance based on the NICE decision. NICE has recommended that autosomal dominant polycystic kidney disease patients in England with chronic kidney disease stages 2 or 3 and evidence of rapidly progressive disease are eligible for treatment. This decision differs from that made by the Scottish Medicines Consortium and the European Medicines Agency in excluding patients with chronic kidney disease stage 1. Evidence of rapid disease progression has been defined as a significant decline in estimated glomerular filtration rate or increase in total kidney volume or both. In the absence of such evidence, risk prediction algorithms based on total kidney volume (Mayo Classification) or genotype (PROPKD) are the best predictors of prognosis. NICE, National Institute for Health and Clinical Excellence.

The approval of tolvaptan by regulatory authorities in Europe and around the world (though not in the USA) for use to slow renal disease progression in ADPKD patients with preserved renal function and evidence of rapidly progressive disease marks a major step-change in the management of this condition. This decision follows the pivotal TEMPO3/4 trial showing significant benefits of treatment on the rate of change in TKV as well as the decline in eGFR ^32^. However, the eligibility criteria for prescribing tolvaptan do vary between countries where approval has been granted ( Table 1). The European Renal Association-European Dialysis and Transplant Association (ERA-EDTA) and the Renal Association have issued more detailed guidance as to what constitutes ‘evidence of rapid disease progression’ so that treatment is offered to patients who are at the highest risk of developing ESRD and therefore potentially the ones to benefit ^33^. The decision pathway published by the Renal Association for the UK is shown in Figure 3 ( http://www.renal.org/guidelines/commentary-on-nice-guidelines#sthash.OrPl3wDl.yCJcc9PF.dpbs). It is clear that the risks and benefits of taking tolvaptan will need to be carefully considered, especially the need for monthly monitoring of liver function tests and the profound aquaretic side-effects requiring high water intake on a daily basis.

**Table 1.** Eligibility criteria for the approved use of tolvaptan according to country or region.

Country	Chronic kidney disease stage	Disease activity	Regulatory body	Approval date	Guidance (if any)
Japan	1–4	TKV >750 ml ΔTKV >5% per annum	Pharmaceuticals and Medical Devices Agency	March 2014	
Canada	Not specified	Not specified	Health Canada	February 2015	
Europe	1–3	Evidence of rapid disease progression	European Medicines Agency	May 2015	European Renal Association-European Dialysis and Transplant Association
England, Wales, and Northern Ireland	2–3	Evidence of rapid disease progression	National Institute for Health and Clinical Excellence (NICE)	October 2015	Renal Association
South Korea	1–3	Evidence of rapid disease progression	Ministry of Food and Drug Safety/Health Insurance Review and Assessment Service	December 2015	
Scotland	1–3	Evidence of rapid disease progression	Scottish Medicines Consortium	January 2016	Renal Association

TKV, total kidney volume.

A number of other drugs, including somatostatin analogues and tyrosine kinase inhibitors, are currently being tested in clinical trials ^1, 34^. Potentially, these could be used in combination with tolvaptan if combined efficacy can be shown clinically as in preclinical models. Many other dietary and therapeutic agents have been shown to have efficacy in preclinical models, although the salutary experience with mammalian target of rapamycin (mTOR) inhibitors has demonstrated the potential divergence between preclinical results and clinical trials ^35, 36^. The experience gained from published trials will improve patient selection, harmonise outcome measures, and likely improve future trial design ^1^.

## A new pathway for managing ADPKD in clinical practice

**Figure 4.**
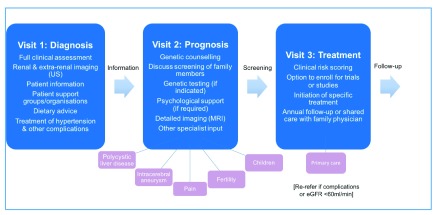
A recommended multidisciplinary stepped pathway for managing patients with autosomal dominant polycystic kidney disease. For simplicity, this has been drawn as a linear follow-up pathway with the major emphasis being on diagnosis, prognosis and treatment at sequential visits. A shared care model with primary care physicians after full evaluation is complete and no treatment planned is an option in nationally funded systems. eGFR, estimated glomerular filtration rate; MRI, magnetic resonance imaging; US, ultrasound.

The rapid progress from gene discovery and understanding of pathophysiology to the first effective treatment for slowing disease progression is changing the management paradigm for ADPKD and stimulating the development of a new patient pathway ( Figure 4) ^1^. Ideally, this should take the form of a multidisciplinary approach beyond measuring renal function and blood pressure. It should consider the management of extra-renal complications (often neglected), which often can give rise to refractory and worrying symptoms ^37– 39^. Family members who are at risk of inheriting the disease should be counselled and offered screening. Psychological and inter-personal issues, including ‘genetic guilt’ (of passing the disease on), are often silent and need to be explored at an early stage ^9^. Patients would benefit from joining local support groups or being part of a patient organisation. Accurate information (clinical, genetic, dietary, or therapeutic) is critical and may be best delivered through nursing or medical staff with expert knowledge. All patients should be offered initial evaluation in specialist PKD clinics with follow-up in secondary or primary care as appropriate to the individual. Many patients (though not all) will wish to be assessed for their eligibility for drug treatment or enrolment in clinical trials. Guidelines for managing patients are being developed in different countries ^40– 42^.

## Conclusions

The management of ADPKD, once considered an untreatable disease, is undergoing a major paradigm shift with more accurate diagnostics, prognostic scoring, and availability of new disease-modifying drugs. This in turn is stimulating a reshaping of patient pathways and the reorganisation of clinical services. A continued focus on developing new therapeutics and renewed attention to patient-centred research priorities are likely to alter the long-term management of ADPKD for patients and their families.
